# Polymorphisms in Pattern Recognition Receptor Genes Are Associated with Respiratory Disease Severity in Pig Farms

**DOI:** 10.3390/ani12223163

**Published:** 2022-11-16

**Authors:** Kasumi Suzuki, Hiroki Shinkai, Gou Yoshioka, Toshimi Matsumoto, Takato Takenouchi, Junji Tanaka, Masanori Shimizu, Haruki Kitazawa, Hirohide Uenishi

**Affiliations:** 1 Swine and Poultry Research Department, Gifu Prefectural Livestock Research Institute, Seki 501-3924, Japan; 2 Food and Feed Immunology Group, Laboratory of Animal Food Function, Graduate School of Agricultural Science, Tohoku University, Sendai 980-8572, Japan; 3 Livestock Immunology Unit, International Education and Research Center for Food Agricultural Immunology (CFAI), Graduate School of Agricultural Science, Tohoku University, Sendai 980-8572, Japan; 4 National Institute of Animal Health, National Agriculture and Food Research Organization (NARO), Tsukuba 305-0856, Japan; 5 Institute of Agrobiological Sciences, National Agriculture and Food Research Organization (NARO), Tsukuba 305-8634, Japan

**Keywords:** pattern recognition receptors, respiratory disease, pleuritis, pneumonia, disease resistance, *Mycoplasma hyopneumoniae*, *Actinobacillus pleuropneumoniae*, single nucleotide polymorphisms, DNA marker, swine

## Abstract

**Simple Summary:**

Infections such as respiratory diseases pose major problems in pig production. We evaluated how these polymorphisms affect pattern recognition receptor genes, altering molecular function and reducing respiratory disease on two pig farms with different health conditions. Three polymorphisms were associated with respiratory disease severity, and they responded differently to two pneumonia pathogens, *Mycoplasma hyopneumoniae,* and *Actinobacillus pleuropneumoniae*, which showed different invasion levels on each farm. Likewise, the respective effects of each polymorphism were more pronounced on the different farms, depending on the severity of the symptoms. These findings demonstrate the possibility of using a combination of immune-gene-located DNA markers to reduce respiratory diseases caused by bacterial pathogens in pig breeding.

**Abstract:**

Reduced productivity caused by infections, particularly respiratory diseases, is a serious problem in pig farming. We have previously reported polymorphisms in porcine pattern recognition receptor genes affecting molecular functions and demonstrated that the 2197A/C polymorphism in the nucleotide-binding oligomerization domain containing 2 (*NOD2*) gene influences porcine circovirus 2-induced mortality. Here, we investigated how these polymorphisms affect respiratory disease-induced lesions, using samples from a slaughterhouse dealing with pigs from two farms. Lung lesions were evaluated using two scoring systems, Goodwin (GW) and slaughterhouse pleuritis evaluation system (SPES), to determine the influence of *Mycoplasma hyopneumoniae* (Mhp) and *Actinobacillus pleuropneumoniae* (App), respectively. SPES scores were significantly higher when the 1205T allele of Toll-like receptor 5 (*TLR5*-1205T), rather than *TLR5*-1205C, was present. On the farm with more severe Mhp invasion, lower GW lesion scores were significantly associated with the presence of the NOD-like receptor family pyrin domain containing 3 (*NLRP3*)-2906G allele; where App invasion was worse, lower SPES scores were significantly associated with the presence of the *NOD2*-2197C allele. Combinations of polymorphisms in pattern recognition receptor genes can therefore be utilized for breeding for resistance against respiratory diseases in pigs. DNA markers of these polymorphisms can thus be used to improve productivity by reducing respiratory diseases due to bacterial pathogens in pig livestock.

## 1. Introduction

In pig farming, infectious diseases pose a constant threat, and infection-related deaths and reduced feed efficiency compromise productivity. Porcine reproductive and respiratory syndrome virus (PRRSV), present in many pig farms, causes respiratory symptoms in growing pigs and impairs reproduction in sows, severely reducing pig production. Bacterial infections, particularly respiratory infections, pose a major threat to pig production. Swine enzootic pneumonia, caused by *Mycoplasma hyopneumoniae*, induces suppression of innate immunity, resulting in secondary infection by viruses and other bacteria [1]. *Actinobacillus pleuropneumoniae* (App) causes pleuritis and pleuropneumonia, causing a huge economic loss in the pig industry [2]. Lung lesions remain after remission of infection; in the slaughterhouse, they can be used to evaluate disease levels and the impact of disease on productivity [3,4,5].

Symptom severity in infectious disease is influenced by the host’s genetic background; this has been especially demonstrated in humans. Mutations in immune genes may affect responses to pathogens, leading to an increase or reduction in susceptibility. In particular, many polymorphisms in pattern recognition receptor (PRR) genes, which constitute a large portion of the innate immune genes involved in recognizing pathogen-derived molecules, are associated with resistance or susceptibility to infectious diseases [6]. We have previously shown that PRR genes contain many polymorphisms, some affecting their recognition of pathogen-derived molecules; this suggests that these polymorphisms can be utilized as DNA markers to improve disease resistance in pigs [7,8].

Polymorphisms in porcine immune genes, including PRR genes, are related to resistance to infection and susceptibility in pigs. We have previously shown that a single nucleotide mutation in *TLR5* (1205C > T), which significantly attenuates the response to *Salmonella*-derived flagellin [9], augments susceptibility to experimental *Salmonella typhimurium* infection, as demonstrated by increased shedding of *Salmonella* in rectal swabs and elevation of the diarrhea score [10]. In pigs, the intracellular PRR nucleotide-binding oligomerization domain containing 1 (*NOD1*) gene has two polymorphic sites, 1922G/A and 2752G/A, that affect ligand recognition [11]. In commercial pig farms, *NOD1*-2752A, which impairs ligand recognition, is significantly associated with increased tissue colonization of *Salmonella* [12]. We have previously examined the relationship between the genotypes of *NOD2* at the 2197A/C locus, which is related to molecular function in pigs [13]. Pigs homozygous for *NOD*-2197C, which augments the response to the ligand, show significantly less mortality due to porcine circovirus 2 (PCV2) [14]. Another intracellular PRR, NOD-like receptor family pyrin domain containing 3 (NLRP3), comprising the NLRP3 inflammasome, which contains apoptosis-associated speck-like protein containing a caspase recruitment domain (ASC) and pro-caspase-1 (CASP1), has a polymorphic site (2906A/G) that is related to mature interleukin-1β production [15]. We have demonstrated that *NLRP3*-2906G, an allele that potentiates an inflammatory response, is associated with increased production of specific antibodies after inoculation with an inactivated vaccine of *Haemophilus parasuis* [16]. These prior findings strongly suggest PRR gene polymorphisms as candidate DNA markers to improve disease resistance in pigs. There is an urgent need to elucidate how such candidate markers influence pig production.

To address this, we investigated the effects of respiratory disease-associated single nucleotide polymorphisms (SNPs) in porcine PRR genes on molecular function. We examined the pathology of lung lesions, which can be detected in slaughterhouses and used as historical indices for infections during growth stages.

## 2. Materials and Methods

### 2.1. Pig Populations

Three-way crossbred ([Landrace × Large White] × Duroc) pigs, maintained in two pig farms (designated as farms A and B) in Gifu Prefecture, Japan, were used in this study. The pigs on farm B were produced on farm A and were transferred at weaning (three weeks after birth). The pigs on both farms, therefore, shared the same genetic background. Farm A pigs were reared in semi-windowless swine barns, which adopted an all-in/all-out production system. In contrast, farm B pigs were reared in open barns, which did not adopt all-in/all-out production at the entire barn level. The pig population is described in Table 1. The pigs were reared in a conventional environment and provided with access to rations and water ad libitum. Male pigs were castrated. Porcine circovirus 2 (PCV2) and *Mycoplasma hyopneumoniae* (Mhp) vaccine FLEXcombo^®^ (Boehringer Ingelheim, Ingelheim am Rhein, Germany) were inoculated into all pigs according to the manufacturer’s instructions. All diets were adapted to the Japanese feeding standard for swine [17]. The reared pigs were shipped to a slaughterhouse when their body weight reached ca. 120 kg (in June 2019–March 2020).

### 2.2. Sampling

Measurements and sampling were conducted in the same slaughterhouse in the Gifu Prefecture. Pigs were sacrificed after overnight lairage without feeding but with free access to water. The pigs were slaughtered by exsanguination after stunning with electricity. The internal organs were separated from the carcasses immediately after slaughter. The carcass and internal organs were cooled in a refrigerator until the lesions were assessed. Muscle tissue was sampled for genomic DNA extraction.

### 2.3. Assessment of Lesions

The pathology of hepatized lung lobe pneumonia lesions was scored as per Goodwin et al. [18], with slight modification. Scoring was conducted in sections, dividing the lung into 55 parts; lesions in each section were evaluated as 0 (none), 0.5 (partly affected), or 1 (totally affected). Goodwin’s lung lesion scores (GW) were logistically transformed to reduce skewness [19,20] as follows:

(1) $$\log\;\mathrm{transformed}\;\mathrm{GW}=\log_{e}\frac{\left(\mathrm{measured}\;\mathrm{GW}+0.5\right)}{\left(55-\mathrm{measured}\;\mathrm{GW}+0.5\right)}+\log_{e}111$$

The last constant was added to adjust the value to zero for samples without lesions. Similarly, pleuropneumonia was evaluated at the slaughterhouse according to the slaughterhouse pleuritis evaluation system (SPES) and scored from 0 (no lesion) to 4 (severely extended lesions, at least one-third of both diaphragmatic lobes) [21,22,23]. Images of typical lung lesions caused by Mhp, and App infections are presented in Figure S1. GW and SPES were evaluated by a trained veterinarian who was a slaughterhouse inspector.

### 2.4. Production-Related Trait Measurement

Carcass backfat thickness (BF) was measured at the thinnest fat area on the 9th to 13th thoracic vertebrae, as per the method of the Japan Meat Grading Association, and adjusted to a carcass weight of 80 kg, as follows:

(2) $$\mathrm{Corrected}\;\mathrm{BF}=\mathrm{measured}\;\mathrm{BF}\times \sqrt[{3}]{\frac{80}{\mathrm{carcass}\;\mathrm{weight}}}$$

The percentage of intramuscular fat (IMF) in the loin portion was assessed between the 4th and 5th intercostals. Antibody responses to Mhp, App and PRRSV were assessed by ELISA, using the IDEXX *M. hyo* Ab Test, IDEXX APP-ApxIV AB Test, and IDEXX PRRS X3 Ab Test, respectively (IDEXX Laboratories, Westbrook, ME, USA). Optical density (OD) was converted into sample-to-positive ratios (S/P) as follows:

(3) $$S/P=\frac{\mathrm{Sample}\;\mathrm{OD}-\mathrm{Negative}\;\mathrm{control}\;\mathrm{OD}}{\mathrm{Positive}\;\mathrm{control}\;\mathrm{OD}-\mathrm{Negative}\;\mathrm{control}\;\mathrm{OD}}$$

We did not observe pigs that were positive for antibody response to PRRSV in either farm A or B; therefore, we considered all of the pigs to be PRRSV-negative and excluded PRRSV antibody production from further analyses (data not shown).

### 2.5. Genotyping

Genotyping of five SNPs in four PRR genes (*NOD1*, *NOD2*, *NLRP3*, and *TLR5*) was conducted using PCR sequencing. PCR and amplicon sequencing was performed as previously described [14] using the listed primers (Table 2). PCR was conducted using AmpliTaq Gold DNA polymerase (Thermo Fisher Scientific, Palo Alto, CA, USA). PCR was initiated by denaturation for 10 min at 94 °C, followed by 45 cycles of 95 °C for 30 s, 55 °C for 30 s, and 72 °C for 1 min. PCR cycles were followed by an additional extension for 5 min at 72 °C. Amplified PCR products were sequenced using an Applied Biosystems 3730xl DNA Analyzer with a BigDye Terminator v3.1 Cycle Sequencing Kit (Thermo Fisher Scientific). The genotypes were confirmed using sequencing reads by automated single nucleotide polymorphism (SNP) detection with PolyPhred [24] and manual inspection using Consed [25] after assembly by Phred basecaller and Phrap assembler [26,27].

### 2.6. Statistical Analysis

Correlations between traits were evaluated using Pearson’s product-moment correlation method. Significant associations between pathogen, immunological, and production-related traits and SNPs in PRR genes and other factors were examined using a generalized linear model (GLM). Traits were assumed to follow a Gaussian distribution. Statistical analyses were performed using R 4.0.5 (https://www.r-project.org/ (accessed on 14 October 2022)).

## 3. Results

### 3.1. Trait Evaluation

GW score was positively and significantly correlated with Mhp antibody production (Table 3). This correlation was marked for farm B, for which both indices were high, but not farm A, which had a lower average GW score (Table 1 and Table S1). SPES score was significantly positively correlated with the App-specific antibody, particularly for farm B (Table 3 and Table S1). For farm A, which had a lower SPES score than farm B, the SPES score and App-specific antibody was not clearly correlated (Table 1 and Table S1). For farm B, GW and SPES scores were significantly correlated (Table S1). Mhp-specific antibody levels were negatively correlated with backfat thickness and positively correlated with IMF content (Table 3). Mhp-specific antibody and IMF were correlated only for farm B (Table S1).

### 3.2. Trait–PRR Polymorphism Associations

All SNPs of the four PRR genes were polymorphic. We have previously shown that the SNP allele distribution differs between the breeds contributing to the pig population (Landrace, Large White, and Duroc); for example, the *TLR5*-1205T allele occurs only in Landrace and Piétrain breeds [11,13,15,29,30,31]. Therefore, the allele distributions did not necessarily conform to the Hardy-Weinberg equilibrium in the populations used (data not shown). The two SNPs in *NOD1*, *NOD1*-1922A/G, and *NOD1*-2752A/G, have a marked effect on NOD1 molecular function, altering its recognition of the peptidoglycan component γ-_D_-glutamyl-*meso*-diaminopimelic acid (iE-DAP) [11]. We there constructed haplotypes of these two *NOD1* SNPs, treating haplotypes with a malfunctioning allele at either SNP site (1922A or 2752A) as negative (*NOD1*^−^). The haplotype with two functional alleles (1922G and 2752G) was considered positive (*NOD1*^+^). We excluded individuals for which we could not definitively determine the *NOD1* haplotype and subsequently used only those with precisely determined genotypes of the four PRR genes (Table 4).

We evaluated the effects of environmental factors on infection- and production-related traits. The GW and SPES scores and Mhp- and App-specific antibody production levels differed significantly between the farms. BF and IMF did not show a significant association with the farm (Table 5). SPES scores and App-specific antibody production levels were associated with slaughter date, with elevated values later in the study.

**Figure 1:**
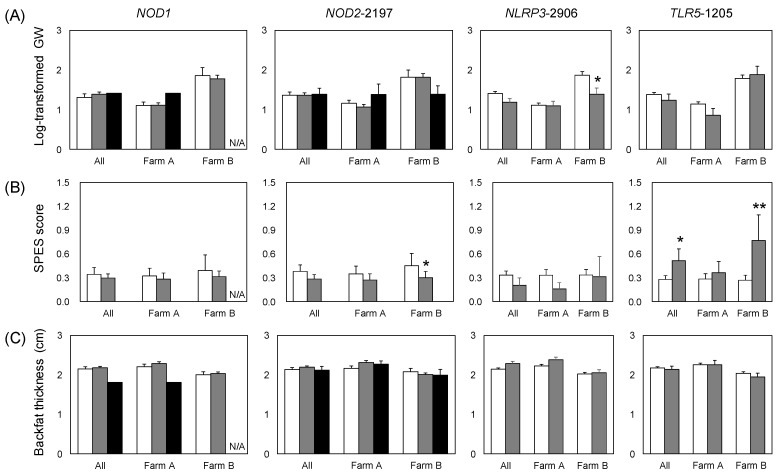

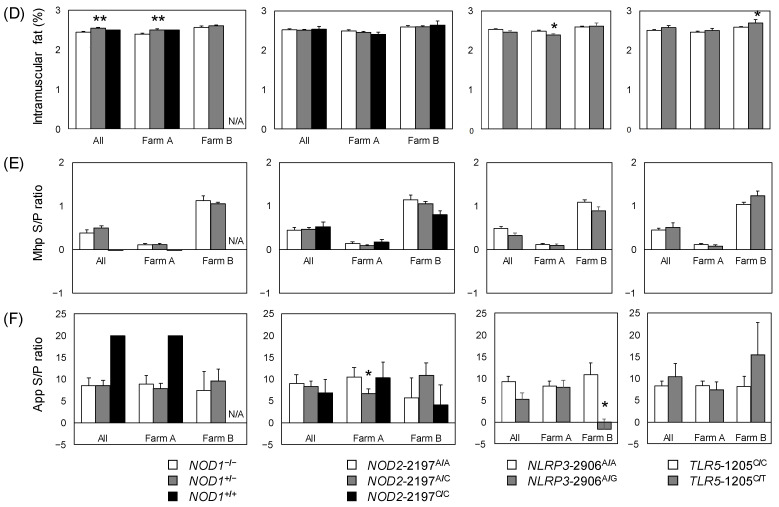
Trait values by PRR genotype for all of the pigs studied. (**A**) log-transformed Goodwin lung lesion score; (**B**) pleuritis (SPES score); (**C**) backfat thickness adjusted by carcass weight; (**D**) intramuscular fat ratio; and antibody titers of (**E**) Mhp and (**F**) App. Error bars: standard error. Significant differences in the estimates (Table 5, Tables S2 and S3) are indicated by asterisks (** *p* < 0.01, * *p* < 0.05). N/A, not applicable, because no *NOD1*^+/+^ individuals were observed on farm B.

We investigated the effects of the PRR genotype on infection-related traits. Further, because the Mhp and App invasion status differed between the farms (Table 1), we assessed variation in the effects of PRR genes between the farms. *NLRP3*-2906 was significantly correlated with the GW score for farm B. The GW score was significantly lower in pigs with the *NLRP3*-2906G allele than in those without it. *NOD2*-2197 and *TLR5*-1205 were significantly associated with the SPES score for farm B. *NOD2*-2197C were significantly negatively correlated with the SPES score, whereas *TLR5*-1205T was significantly positively correlated with it (Figure 1, Table S3). *TLR5*-1205 and SPES scores were significantly associated with all pigs combined from both farms (Figure 1, Table 5). Mhp-specific antibody production was not significantly associated with the PRR genotype. App-specific antibody production was significantly negatively associated with *NOD2*-2197C for farm A and *NLRP3*-2906G for farm B (Figure 1, Tables S2 and S3).

We evaluated associations between PRR genotype and production-related traits (BF and IMF). Comparing the alleles, IMF was significantly higher in *NOD1*^+^ individuals on farm A and for the farms combined (Figure 1, Table 1 and Table S2). On farm B, IMF was significantly higher in pigs with the *TLR5*-1205T allele than in those with *TLR5*-1205C (Figure 1 and Table S3).

## 4. Discussion

We analyzed associations between PRR-gene SNPs and phenotype in pigs from two farms, particularly in terms of disease-related traits and immunological indices. The observed severity of pathogenic invasion highlights the value of SNPs as disease-resistant DNA markers.

The GW and SPES lung lesion scores and Mhp- and App-specific antibody titers were significantly higher for farm B than farm A, and farm B exhibited clear correlations between the lung lesion scores and antibody levels. These correlations were not significant for farm A. Significant correlations between the lung lesion scores and antibody titers indicate that the bacterial agents caused the increases in the lung lesion scores. As the genetic backgrounds of the pigs in the two farms were identical, the differences in the infection status between the farms plausibly reflected the differences in the rearing environments.

These pigs were inoculated with the Mhp vaccine just after weaning. Mhp vaccination elicits a weak (and typically negative) antibody response [32], as in our results. The significantly higher Mhp-specific antibody response on farm B indicates that this response was due to natural infection and reflects the severity of Mhp invasion. App vaccination was not applied on either farm; hence the App-specific antibody response was also due to natural infection. Although SPES scores, which primarily reflect App infection, are distinctly correlated with App antigen levels [33], they may also reflect other infections, such as PRRS [22]. Here, although the population was negative for PRRSV, other pathogens might have influenced the SPES score on farm A, which had a relatively low average SPES score.

We found that PRR-gene SNPs were associated with infection-related indices, particularly for farm B, where severe Mhp and App infections were observed. The *TLR5*-1205T allele reduces the recognition ability of flagellin, a protein found in bacterial flagella [9]. On farm B, the presence of this allele significantly increased the SPES score. Although the same tendency was observed on farm A, the difference in SPES score was not significant, probably because of the low severity of symptoms. We have previously shown an association between *TLR5* genotype and App-vaccination antibody response. In that study, only synonymous *TLR5* polymorphisms were present in the pig population; however, a particular genotype comprising these synonymous polymorphisms was significantly associated with App-specific antibody production [34]. This suggests that *TLR5* expression enhances the App-specific antibody response to this bacterium, which is flagellated under particular environmental conditions [35]. The nonsynonymous polymorphism *TLR5*-1205, related to flagellin recognition, might be involved in the eradication of App in natural infection and might have influenced the pleuritis that we observed here.

The *NOD2*-2197C allele provides improved recognition of muramyldipeptide, a component of peptidoglycan [13]. This allele significantly reduced the SPES score on farm B. The *NOD2*-2197C allele might enhance the response to the gram-negative bacteria *A. pleuropneumoniae* cell body [36], thus mitigating pleuritis symptoms. Infections with pathogens other than App should be considered when SPES scores are high. These findings reveal that enhanced NOD2 function is widely effective in preventing infection by peptidoglycan-containing bacteria and effectively reduces SPES scores.

*Mycoplasma hyopneumoniae* belongs to the class *Mollicutes*, gram-positive bacteria lacking a peptidoglycan layer and flagellae [37]. It, therefore, escapes immune surveillance by TLR5 and NOD2. This might explain why pigs with the *TLR5*-1205CT or *NOD2*-2197AC genotypes did not show distinctly different GW scores from those with the *TLR5*-1205CC or *NOD2*-2197AA genotypes, respectively. On farm B, the *NOD2*-2197CC genotype was associated with lower GW and SPES scores than the other genotypes, although nonsignificantly (possibly owing to the low number of *NOD2*-2197CC individuals). Pathogens other than Mhp may aggravate mycoplasmal pneumonia. This suggests that NOD2 and other PRR-related molecules should be considered as part of a strategy to reduce the GW score in pigs.

The SNP at *NLRP3*-2906 significantly affected the GW score. On farm B, GW scores were lowest among individuals with the *NLRP3*-2906G genotype. This polymorphism augments the specific antibody response to inactivated vaccines, presumably because the *NLRP3*-inflammasome enhances the inflammatory response [15,16]. On farm B, we found that the *NLRP3*-2906G polymorphism significantly reduced the GW score relative to the other genotypes. Here, individuals with the *NLRP3*-2906G genotype did not exhibit potentiation of the specific antibody response to Mhp vaccination, suggesting that the reduction in GW score was not directly due to *NLRP3*-2906G-induced enhancement of Mhp-vaccination. In vitro and in vivo studies using a mouse model have revealed NLRP3 as a critical factor in increasing inflammation in *M. pneumoniae* infection [38]. These results suggest that enhancement of NLRP3 function ameliorates mycoplasmal pneumonia by suppressing secondary infection by other pathogens.

On farm B, the App-specific antibody response was significantly lower for the *NLRP3*-2906AG genotype than for the *NLRP3*-2906AA genotype, suggesting that *NLRP3*-2906G might contribute to inhibiting infections such as App. Mhp infection induces immunosuppressive processes such as phagocytosis by polymorphonuclear neutrophils, thus enhancing secondary infection [39]. Death due to mycoplasmal pneumonia is usually the result of deterioration of symptoms due to secondary infection [40]. PRR SNPs that are not involved in Mhp recognition may therefore have important roles in resistance to mycoplasmal pneumonia. Many pathogens, including bacteria, fungi, and viruses, are associated with secondary infection. While immune-related gene SNPs can ameliorate mycoplasmal pneumonia, the types of SNPs found on farms will vary depending on the types of invading pathogens present. In particular, further research is required into the roles of *TLR2* or *TLR6* polymorphisms in pneumonia. We have previously shown that TLR2 and TLR6 participate directly in Mhp recognition [41].

Lipid-related traits, such as BF and IMF, did not differ markedly between the farms or individuals. However, the *NOD1*-1922G/2752G alleles significantly increased IMF on farm A relative to that of the other genotypes. In contrast, on farm B, individuals with the *TLR5*-1205T allele showed significantly more IMF. NOD1, which markedly affects the intestinal flora, is related to the onset of inflammatory bowel diseases in humans [42]. Mice lacking *TLR5* showed alterations in the bowel flora, exhibiting symptoms such as metabolic syndrome and increased adiposity [43]. NOD1 and TLR5 may affect lipid metabolism by altering the intestinal microbiota. Here, on farm A, *NLRP3*-2906G individuals showed significantly lower IMF than those with the other genotypes, whereas, on farm B, this genotype showed (nonsignificantly) higher IMF. The influence of PRR-gene polymorphisms on lipid-related traits remains unclear and requires further investigation. This also applies also to NOD2, which profoundly affects the intestinal microbiota in humans; its polymorphisms are related to Crohn’s disease [44,45].

In the Japanese pork market, production-related traits such as IMF are important in terms of consumer appeal and pricing. Even if PRR-gene polymorphisms themselves are not related to these traits, other genes in their vicinity may be. There is, therefore, a need for further examination of the effects of improved genetic disease resistance on IMF and other production-related traits.

Effects of the disease-resistant DNA markers may be evaluated more efficiently by experimental infection rather than investigating pig farms with natural infection, as in the present study. In studies with experimental infection, variations in antibody response and/or cytokine production can be assessed in a time-course manner, which may allow for investigating the relationship between such immune responses and alleles of the DNA markers. However, the significance of the present study lies in demonstrating the usefulness of DNA markers in suppressing onset of respiratory diseases in pig farms with natural infections of App and Mhp, which can be extrapolated to ordinary farms.

Unlike the cellular receptors involved in pathogenic infection, PRRs do not exhibit high specificity to pathogens, rather protecting against a wide variety of pathogens. Here, we examined PRR SNP responses specifically to Mhp and App. However, these PRRs may also respond to other bacteria or to viruses, protecting against secondary infection, as suggested for PCV2 [14]. Further investigation is required to identify combinations of immune-gene DNA markers that are effective against infectious diseases in swine, including viral infections, to accelerate pig breeding and improve disease resistance.

## 5. Conclusions

In pigs from two farms, we investigated the association between infection-related phenotypes and PRR-gene SNPs known to affect molecular function. Mhp and App invasions on the farms were associated with specific SNPs, symptoms, and specific antibody responses. *NOD2* and *TLR5* SNPs were associated with pleuritis, whereas *NLRP3* SNPs were associated with an index of mycoplasmal pneumonia. Pathogen invasion varies by farm, and the associated effective PRR SNPs may change. Therefore, DNA marker sets comprising combinations of these SNPs may hold promise for improving disease resistance in pig populations.

## Figures and Tables

**Table 1:** Pig populations were used in this study. Upper and lower values correspond to farms A and B, respectively.

Trait		Unit	N		Mean ± SE	Positive/Negative Animals ^1^
				Sex (Male/Female)		
GW lung lesion score (log-transformed)		-	180 106	(99/81) (49/57)	1.110 ± 0.050 1.797 ± 0.081	142/38 100/6
SPES pleuritis score		-	180 106	(99/81) (49/57)	0.294 ± 0.060 0.330 ± 0.069	29/151 26/80
Backfat thickness (adjusted with carcass weight)		cm	180 106	(99/81) (49/57)	2.259 ± 0.038 2.030 ± 0.035	-
Intramuscular fat (IMF)		%	171 106	(95/76) (49/57)	2.465 ± 0.020 2.596 ± 0.020	-
Antibody	Mhp	-	180 103	(99/81) (48/55)	0.111 ± 0.015 1.062 ± 0.042	8/172 98/5
	App	-	180 103	(99/81) (48/55)	8.186 ± 1.035 9.040 ± 2.341	2/178 6/97

GW, Goodwin; SPES, slaughterhouse pleuritis evaluation system; Mhp, *Mycoplasma hyopneumoniae;* App, *Actinobacillus pleuropneumoniae*. SE, standard error. ^1^ Individuals with no lesions based on GW or SPES scoring were designated as negative. Antibody production was assessed as positive via ELISA for Mhp > 0.4 and App ≥ 50, as per the manufacturer’s instructions.

**Table 2:** Genotyped SNPs in PRR genes and primer was used.

Gene	SNP Position	Location on Pig Genome ^1^		Allele ^2^	Primer Sequences
		Chromosome	Position		
*NLRP3*	2906	2	56,897,226	A/G	5′-CCAAGCTTGTTAATCTTGTGC-3′ 5′-AAGTGCAAATGAAGCCATCC-3′
*NOD1*	1922	18	42,478,230	A/G	5′-GTCCAAAGGCAAACAGAAACTC-3′ 5′-GAGAAGGTCTGGATGTTCCAAG-3′
	2752	18	42,496,337	A/G	5′-CTGGTGGTCTCCAAACCATT-3′ 5′-TCCACATCTGCGAAACAGAG-3′
*NOD2*	2197	6	34,157,241	A/C	5′-GGTGTCTGAGAAGGCTCTGC-3′ 5′-TTGCAGACGTTGAGACAAGG-3′
*TLR5*	1205	10	19,391,571	T/C	5′-TCTGGGTTTGGCTTCCATAA-3′ 5′-TCAGATGGCGAAAGACTCCT-3′

^1^ Locations on chromosomes and nucleotide positions in the pig reference genome sequence (Sscrofa11.1 [28]) are indicated. ^2^ The letter on the right reflects the allele augmenting the molecular function of the gene product.

**Table 3:** Pearson correlation coefficients between the traits evaluated.

Traits	SPES	BF	IMF	Ab (Mhp)	Ab (App)
GW	0.115 (0.052)	−0.066 (0.264)	0.117 (0.052)	0.483 (<0.001 ***)	0.003 (0.956)
	SPES	−0.065 (0.272)	0.124 (0.039 *)	0.077 (0.195)	0.134 (0.024 *)
		BF	−0.048 (0.423)	−0.189 (0.001 **)	−0.106 (0.076)
			IMF	0.263 (<0.001 ***)	0.063 (0.299)
				Ab (Mhp)	0.091 (0.127)

GW, Goodwin lung lesion score (log-transformed); SPES, slaughterhouse pleuritis evaluation system; BF, backfat thickness; IMF, intramuscular fat; Ab, antibody; Mhp, *Mycoplasma hyopneumoniae*; App, *Actinobacillus pleuropneumoniae*. *** *p* < 0.001; ** *p* < 0.01; * *p* < 0.05.

**Table 4:** Allele distribution of PRR genes in the pig populations.

Farm	*NOD1* Haplotype ^1^			*NOD2*-2197			*NLRP3*-2906			*TLR5*-1205		
	−/−	+/−	+/+	AA	AC	CC	AA	AG	GG	CC	CT	TT
A	62	117	1	69	107	4	142	38	0	158	22	0
B	23	83	0	31	70	5	90	16	0	93	13	0

^1^ Reconstructed haplotypes of *NOD1* SNPs combined with *NOD1*-1922 and *NOD1*-2752. Minus (−) indicates a haplotype with at least *NOD1*-1922A or *NOD1*-2752A. Plus (+) indicates haplotypes *NOD1*-1922G and *NOD1*-2752G. Individuals in which *NOD1* haplotypes could not be determined were omitted from the analysis.

**Table 5:** PRR genotype–trait associations based on GLM analysis. (A) Goodwin lung lesion score; (B) SPES pleuritis lesion score; (C) backfat thickness; (D) intramuscular fat; and antibodies specific to (E) Mhp and (F) App. Farm, sex, and slaughter date were included as factors. The single *NOD1*^+/+^ haplotype individual detected was excluded from the analysis. *** *p* < 0.001; ** *p* < 0.01; * *p* < 0.05.

**(A) Goodwin Lung Lesion Score (GW)**							
**Reference Group**	**Tested Group**	**Coefficient**	**SE**	**Confidence Interval (≥95%)**		**Hypothesis Test**	
				**Lower**	**Upper**	***t***	***p***
Farm (A)	B	1.059	0.267	0.535	1.583	3.961	<0.001 ***
Sex (Male)	Female	0.018	0.088	−0.155	0.191	0.206	0.837
*NOD1* (−/−)	+/−	−0.019	0.097	−0.210	0.172	−0.195	0.845
*NOD2*-2197 (A/A)	A/C	−0.056	0.095	−0.242	0.130	−0.588	0.556
	C/C	−0.195	0.260	−0.705	0.314	−0.752	0.452
*NLRP3*-2906 (A/A)	A/G	−0.181	0.113	−0.402	0.040	−1.603	0.109
*TLR5*-1205 (C/C)	C/T	−0.132	0.135	−0.397	0.133	−0.975	0.329
Date		0.002	0.002	−0.001	0.005	1.481	0.139
Intercept		−40.941	28.445	−96.693	14.810	−1.439	0.150
**(B) Pleuritis-lesion score (SPES)**							
**Reference Group**	**Tested Group**	**Coefficient**	**SE**	**Confidence interval (≥95%)**		**Hypothesis test**	
				**Lower**	**Upper**	***t***	***p***
Farm (A)	B	1.110	0.273	0.575	1.645	4.068	<0.001 ***
Sex (Male)	Female	0.022	0.090	−0.155	0.198	0.242	0.809
*NOD1* (−/−)	+/−	−0.028	0.099	−0.223	0.167	−0.280	0.779
*NOD2*-2197 (A/A)	A/C	−0.167	0.097	−0.357	0.022	−1.731	0.084
	C/C	−0.436	0.265	−0.956	0.083	−1.646	0.100
*NLRP3*-2906 (A/A)	A/G	−0.177	0.115	−0.403	0.049	−1.537	0.124
*TLR5*-1205 (C/C)	C/T	0.279	0.138	0.009	0.550	2.025	0.043 *
Date		0.007	0.002	0.003	0.010	4.147	<0.001 ***
Intercept		−119.964	29.020	−176.842	−63.086	−4.134	<0.001 ***
**(C) Backfat thickness**							
**Reference Group**	**Tested Group**	**Coefficient**	**SE**	**Confidence interval (≥95%)**		**Hypothesis test**	
				**Lower**	**Upper**	***t***	***p***
Farm (A)	B	0.123	0.165	−0.199	0.446	0.748	0.454
Sex (Male)	Female	−0.092	0.054	−0.198	0.015	−1.689	0.091
*NOD1* (−/−)	+/−	0.046	0.060	−0.071	0.163	0.768	0.443
*NOD2*-2197 (A/A)	A/C	0.053	0.058	−0.061	0.168	0.915	0.360
	C/C	0.033	0.160	−0.280	0.347	0.210	0.834
*NLRP3*-2906 (A/A)	A/G	0.091	0.069	−0.045	0.227	1.308	0.191
*TLR5*-1205 (C/C)	C/T	−0.036	0.083	−0.199	0.127	−0.432	0.666
Date		0.002	0.001	0.000	0.004	2.272	0.023 *
Intercept		−37.559	17.505	−71.869	−3.250	−2.146	0.032 *
**(D) Intramuscular fat**							
**Reference Group**	**Tested Group**	**Coefficient**	**SE**	**Confidence interval (≥95%)**		**Hypothesis test**	
				**Lower**	**Upper**	***t***	***p***
Farm (A)	B	0.058	0.088	−0.114	0.231	0.663	0.507
Sex (Male)	Female	−0.019	0.029	−0.077	0.038	−0.661	0.509
*NOD1* (−/−)	+/−	0.097	0.033	0.033	0.161	2.965	0.003 **
*NOD2*-2197 (A/A)	A/C	−0.030	0.032	−0.091	0.032	−0.933	0.351
	C/C	−0.049	0.085	−0.216	0.119	−0.569	0.569
*NLRP3*-2906 (A/A)	A/G	−0.060	0.038	−0.133	0.014	−1.592	0.111
*TLR5*-1205 (C/C)	C/T	0.081	0.045	−0.008	0.169	1.791	0.073
Date		0.000	0.001	−0.001	0.001	−0.744	0.457
Intercept		9.398	9.354	−8.936	27.732	1.005	0.315
**(E) Mhp-specific antibodies**							
**Reference Group**	**Tested Group**	**Coefficient**	**SE**	**Confidence interval (≥95%)**		**Hypothesis test**	
				**Lower**	**Upper**	***t***	***p***
Farm (A)	B	0.849	0.110	0.633	1.065	7.703	<0.001 ***
Sex (Male)	Female	0.117	0.036	0.046	0.188	3.228	0.001 **
*NOD1* (−/−)	+/−	−0.001	0.040	−0.079	0.077	−0.031	0.975
*NOD2*-2197 (A/A)	A/C	−0.054	0.039	−0.131	0.022	−1.394	0.163
	C/C	−0.193	0.106	−0.401	0.015	−1.820	0.069
*NLRP3*-2906 (A/A)	A/G	−0.064	0.046	−0.155	0.027	−1.371	0.170
*TLR5*-1205 (C/C)	C/T	0.055	0.055	−0.053	0.163	0.993	0.321
Date		−0.001	0.001	−0.002	0.001	−0.922	0.356
Intercept		10.867	11.668	−12.001	33.736	0.931	0.352
**(F) App-specific antibodies**							
**Reference Group**	**Tested Group**	**Coefficient**	**SE**	**Confidence interval (≥95%)**		**Hypothesis test**	
				**Lower**	**Upper**	***t***	***p***
Farm (A)	B	20.968	6.561	8.110	33.827	3.196	0.001 **
Sex (Male)	Female	−2.798	2.153	−7.018	1.422	−1.300	0.194
*NOD1* (−/−)	+/−	0.057	2.367	−4.582	4.696	0.024	0.981
*NOD2*-2197 (A/A)	A/C	−1.809	2.317	−6.351	2.732	−0.781	0.435
	C/C	−3.242	6.313	−15.615	9.131	−0.514	0.608
*NLRP3*-2906 (A/A)	A/G	−4.934	2.765	−10.355	0.486	−1.784	0.074
*TLR5*-1205 (C/C)	C/T	2.790	3.286	−3.651	9.231	0.849	0.396
Date		0.123	0.038	0.048	0.197	3.234	0.001 **
Intercept		−2235.554	694.545	−3596.838	−874.270	−3.219	0.001 **

## Data Availability

The data presented in this study are available on request from the corresponding authors.

## Supplementary Materials

The following supporting information can be downloaded at https://www.mdpi.com/article/10.3390/ani12223163/s1: Figure S1: Representative images of lung lesions caused by two respiratory infections. Table S1: Pearson correlation coefficients between the traits evaluated for pig farms A and B; Table S2: PRR genotype–trait associations based on GLM analysis for farm A; Table S3: PRR genotype–trait associations based on GLM analysis for farm B.

## References

[1] Wang H., Zhang Z., Xie X., Liu B., Wei Y., Gan Y., Yuan T., Ni B., Wang J., Zhang L. (2020). Paracellular pathway-mediated *Mycoplasma hyopneumoniae* migration across porcine airway epithelial barrier under air-liquid interface conditions. Infect. Immun..

[2] Losinger W.C. (2005). Economic impacts of reduced pork production associated with the diagnosis of *Actinobacillus pleuropneumoniae* on grower/finisher swine operations in the United States. Prev. Vet. Med..

[3] Bossé J.T., Janson H., Sheehan B.J., Beddek A.J., Rycroft A.N., Kroll J.S., Langford P.R. (2002). *Actinobacillus pleuropneumoniae*: Pathobiology and pathogenesis of infection. Microbes Infect..

[4] Ferraz M.E.S., Almeida H.M.S., Storino G.Y., Sonálio K., Souza M.R., Moura C.A.A., Costa W.M.T., Lunardi L., Linhares D.C.L., de Oliveira L.G. (2020). Lung consolidation caused by *Mycoplasma hyopneumoniae* has a negative effect on productive performance and economic revenue in finishing pigs. Prev. Vet. Med..

[5] Sánchez P., Pallarés F.J., Gómez M.A., Bernabé A., Gómez S., Seva J. (2018). Importance of the knowledge of pathological processes for risk-based inspection in pig slaughterhouses (Study of 2002 to 2016). Asian-Australas. J. Anim. Sci..

[6] Skevaki C., Pararas M., Kostelidou K., Tsakris A., Routsias J.G. (2015). Single nucleotide polymorphisms of Toll-like receptors and susceptibility to infectious diseases. Clin. Exp. Immunol..

[7] Uenishi H., Shinkai H., Morozumi T., Muneta Y. (2012). Genomic survey of polymorphisms in pattern recognition receptors and their possible relationship to infections in pigs. Vet. Immunol. Immunopathol..

[8] Uenishi H., Shinkai H. (2009). Porcine Toll-like receptors: The front line of pathogen monitoring and possible implications for disease resistance. Dev. Comp. Immunol..

[9] Shinkai H., Suzuki R., Akiba M., Okumura N., Uenishi H. (2011). Porcine Toll-like receptors: Recognition of *Salmonella enterica* serovar Choleraesuis and influence of polymorphisms. Mol. Immunol..

[10] Muneta Y., Arai N., Yakabe Y., Eguchi M., Shibahara T., Sakuma A., Shinkai H., Uenishi H., Hirose K., Akiba M. (2018). *In vivo* effect of a TLR5 SNP (C1205T) on *Salmonella enterica* serovar Typhimurium infection in weaned, specific pathogen-free Landrace piglets. Microbiol. Immunol..

[11] Shinkai H., Matsumoto T., Toki D., Okumura N., Terada K., Uenishi H. (2015). Porcine NOD1 polymorphisms with impaired ligand recognition and their distribution in pig populations. Mol. Immunol..

[12] Ainslie-Garcia M.H., Farzan A., Jafarikia M., Lillie B.N. (2018). Single nucleotide variants in innate immune genes associated with *Salmonella* shedding and colonization in swine on commercial farms. Vet. Microbiol..

[13] Jozaki K., Shinkai H., Tanaka-Matsuda M., Morozumi T., Matsumoto T., Toki D., Okumura N., Eguchi-Ogawa T., Kojima-Shibata C., Kadowaki H. (2009). Influence of polymorphisms in porcine NOD2 on ligand recognition. Mol. Immunol..

[14] Suzuki K., Shinkai H., Yoshioka G., Matsumoto T., Tanaka J., Hayashi N., Kitazawa H., Uenishi H. (2021). NOD2 genotypes affect the symptoms and mortality in the porcine circovirus 2-spreading pig population. Genes.

[15] Tohno M., Shinkai H., Toki D., Okumura N., Tajima K., Uenishi H. (2016). Identification of the Q969R gain-of-function polymorphism in the gene encoding porcine NLRP3 and its distribution in pigs of Asian and European origin. Immunogenetics.

[16] Shinkai H., Terada K., Toki D., Tohno M., Uenishi H. (2018). Q969R polymorphism in NLRP3 is associated with immune responses to vaccination against bacterial infections in pigs. Anim. Sci. J..

[17] National Agriculture and Food Research Organization (2013). Japanese Feeding Standard for Swine.

[18] Goodwin R.F., Whittlestone P. (1973). Enzootic pneumonia of pigs: Immunization attempts inoculating *Mycoplasma suipneumoniae* antigen by various routes and with different adjuvants. Br. Vet. J..

[19] Djordjevic S.P., Eamens G.J., Romalis L.F., Nicholls P.J., Taylor V., Chin J. (1997). Serum and mucosal antibody responses and protection in pigs vaccinated against *Mycoplasma hyopneumoniae* with vaccines containing a denatured membrane antigen pool and adjuvant. Aust. Vet. J..

[20] Okamura T., Onodera W., Tayama T., Kadowaki H., Kojima-Shibata C., Suzuki E., Uemoto Y., Mikawa S., Hayashi T., Awata T. (2012). A genome-wide scan for quantitative trait loci affecting respiratory disease and immune capacity in Landrace pigs. Anim. Genet..

[21] Fraile L., Alegre A., López-Jiménez R., Nofrarías M., Segalés J. (2010). Risk factors associated with pleuritis and cranio-ventral pulmonary consolidation in slaughter-aged pigs. Vet. J..

[22] Merialdi G., Dottori M., Bonilauri P., Luppi A., Gozio S., Pozzi P., Spaggiari B., Martelli P. (2012). Survey of pleuritis and pulmonary lesions in pigs at abattoir with a focus on the extent of the condition and herd risk factors. Vet. J..

[23] Sibila M., Aragón V., Fraile L., Segalés J. (2014). Comparison of four lung scoring systems for the assessment of the pathological outcomes derived from *Actinobacillus pleuropneumoniae* experimental infections. BMC Vet. Res..

[24] Nickerson D.A., Tobe V.O., Taylor S.L. (1997). PolyPhred: Automating the detection and genotyping of single nucleotide substitutions using fluorescence-based resequencing. Nucleic Acids Res..

[25] Gordon D., Abajian C., Green P. (1998). Consed: A graphical tool for sequence finishing. Genome Res..

[26] Ewing B., Green P. (1998). Base-calling of automated sequencer traces using *Phred*. II. Error probabilities. Genome Res..

[27] Ewing B., Hillier L., Wendl M.C., Green P. (1998). Base-calling of automated sequencer traces using *Phred*. I. Accuracy assessment. Genome Res..

[28] Groenen M.A., Archibald A.L., Uenishi H., Tuggle C.K., Takeuchi Y., Rothschild M.F., Rogel-Gaillard C., Park C., Milan D., Megens H.J. (2012). Analyses of pig genomes provide insight into porcine demography and evolution. Nature.

[29] Kojima-Shibata C., Shinkai H., Morozumi T., Jozaki K., Toki D., Matsumoto T., Kadowaki H., Suzuki E., Uenishi H. (2009). Differences in distribution of single nucleotide polymorphisms among intracellular pattern recognition receptors in pigs. Immunogenetics.

[30] Muneta Y., Minagawa Y., Kusumoto M., Shinkai H., Uenishi H., Splichal I. (2012). Allele-specific primer polymerase chain reaction for a single nucleotide polymorphism (C1205T) of swine toll-like receptor 5 and comparison of the allelic frequency among several pig breeds in Japan and the Czech Republic. Microbiol. Immunol..

[31] Shinkai H., Tanaka M., Morozumi T., Eguchi-Ogawa T., Okumura N., Muneta Y., Awata T., Uenishi H. (2006). Biased distribution of single nucleotide polymorphisms (SNPs) in porcine Toll-like receptor 1 (*TLR1*), *TLR2*, *TLR4*, *TLR5*, and *TLR6* genes. Immunogenetics.

[32] Lee S.I., Jeong C.G., Ul Salam Mattoo S., Nazki S., Prasad Aganja R., Kim S.C., Khatun A., Oh Y., Noh S.H., Lee S.M. (2021). Protective immunity induced by concurrent intradermal injection of porcine circovirus type 2 and *Mycoplasma hyopneumoniae* inactivated vaccines in pigs. Vaccine.

[33] Liao S., Lee J., Chen F., Lee W., Wu Y., Hsuan S., Kuo C., Chang Y., Chen T. (2017). Evaluation of lung scoring system and serological analysis of *Actinobacillus pleuropneumoniae* infection in pigs. Pak. Vet. J..

[34] Shinkai H., Arakawa A., Tanaka-Matsuda M., Ide-Okumura H., Terada K., Chikyu M., Kawarasaki T., Ando A., Uenishi H. (2012). Genetic variability in swine leukocyte antigen class II and Toll-like receptors affects immune responses to vaccination for bacterial infections in pigs. Comp. Immunol. Microbiol. Infect. Dis..

[35] Negrete-Abascal E., Reyes M.E., García R.M., Vaca S., Girón J.A., García O., Zenteno E., De La Garza M. (2003). Flagella and motility in *Actinobacillus pleuropneumoniae*. J. Bacteriol..

[36] Rycroft A.N., Garside L.H. (2000). *Actinobacillus* species and their role in animal disease. Vet. J..

[37] Miyata M., Hamaguchi T. (2016). Prospects for the gliding mechanism of *Mycoplasma mobile*. Curr. Opin. Microbiol..

[38] Segovia J.A., Chang T.H., Winter V.T., Coalson J.J., Cagle M.P., Pandranki L., Bose S., Baseman J.B., Kannan T.R. (2018). NLRP3 is a critical regulator of inflammation and innate immune cell response during *Mycoplasma pneumoniae* infection. Infect. Immun..

[39] Asai T., Okada M., Yokomizo Y., Sato S., Mori Y. (1996). Suppressive effect of bronchoalveolar lavage fluid from pigs infected with *Mycoplasma hyopneumoniae* on chemiluminescence of porcine peripheral neutrophils. Vet. Immunol. Immunopathol..

[40] Baskerville A. (1981). Pneumonia of pigs: A review. N. Z. Vet. J..

[41] Muneta Y., Uenishi H., Kikuma R., Yoshihara K., Shimoji Y., Yamamoto R., Hamashima N., Yokomizo Y., Mori Y. (2003). Porcine TLR2 and TLR6: Identification and their involvement in *Mycoplasma hyopneumoniae* infection. J. Interferon Cytokine Res..

[42] McGovern D.P., Hysi P., Ahmad T., van Heel D.A., Moffatt M.F., Carey A., Cookson W.O., Jewell D.P. (2005). Association between a complex insertion/deletion polymorphism in *NOD1* (*CARD4*) and susceptibility to inflammatory bowel disease. Hum. Mol. Genet..

[43] Vijay-Kumar M., Aitken J.D., Carvalho F.A., Cullender T.C., Mwangi S., Srinivasan S., Sitaraman S.V., Knight R., Ley R.E., Gewirtz A.T. (2010). Metabolic syndrome and altered gut microbiota in mice lacking Toll-like receptor 5. Science.

[44] Hugot J.P., Chamaillard M., Zouali H., Lesage S., Cézard J.P., Belaiche J., Almer S., Tysk C., O’Morain C.A., Gassull M. (2001). Association of NOD2 leucine-rich repeat variants with susceptibility to Crohn’s disease. Nature.

[45] Ogura Y., Bonen D.K., Inohara N., Nicolae D.L., Chen F.F., Ramos R., Britton H., Moran T., Karaliuskas R., Duerr R.H. (2001). A frameshift mutation in *NOD2* associated with susceptibility to Crohn’s disease. Nature.

