# Supplementary material for: Polymorphisms in Pattern Recognition Receptor Genes Are Associated with Respiratory Disease Severity in Pig Farms
Source: Animals (Basel). 2022 Nov 16;12(22):3163. doi: 10.3390/ani12223163 (PMC9686681; doi:10.3390/ani12223163)
Supplement: Supplementary file 1 [file animals-12-03163-s001.zip › animals-2000274-supplementary.pdf]

(A)

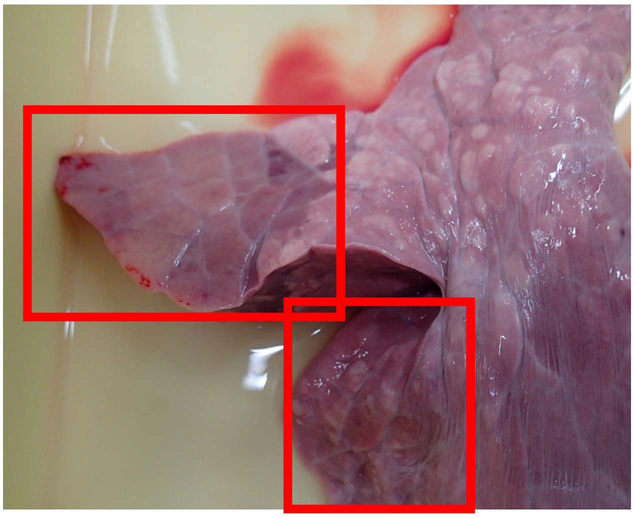

(B)

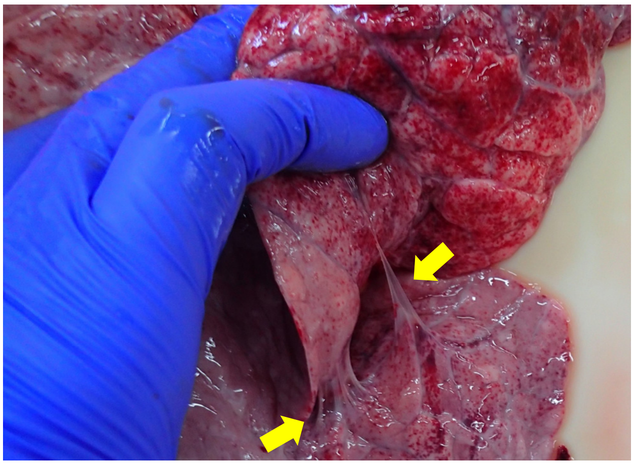

**Figure S1.** Representative images of lung lesions caused by two respiratory infections. (A) Hepatized lung lesions typically caused by *Mycoplasma hyopneumoniae* infection. Hepatized regions in the lung lobes, indicated by red rectangles, were determined as lesions, according to the Goodwin lung lesion scoring system. (B) Representative images of lung lesions typically caused by *Actinobacillus pleuropneumoniae* infection. Adhesions between the lobes, used for evaluating the slaughterhouse pleuritis evaluation system score, are indicated by yellow arrows.

**Table S1.** Pearson correlation coefficients between the traits evaluated for pig farms A and B.

**(A)** Traits of pigs in farm A.

| **Traits** | **SPES** | **BF** | **IMF** | **Ab (Mhp)** | **Ab (App)** |
|---|---|---|---|---|---|
| **GW** | 0.043 (0.568) | 0.106 (0.159) | −0.037 (0.634) | −0.083 (0.269) | −0.049 (0.511) |
| | **SPES** | −0.032 (0.672) | 0.142 (0.062) | 0.088 (0.242) | 0.074 (0.321) |
| | | **BF** | −0.032 (0.678) | 0.020 (0.794) | −0.044 (0.556) |
| | | | **IMF** | 0.024 (0.757) | 0.038 (0.618) |
| | | | | **Ab (Mhp)** | 0.333 (<0.001***) |

(B) Traits of pigs in farm B.

| **Traits** | **SPES** | **BF** | **IMF** | **Ab (Mhp)** | **Ab (App)** |
|---|---|---|---|---|---|
| **GW** | 0.233 (0.016*) | −0.095 (0.332) | 0.098 (0.315) | 0.534 (<0.001***) | 0.028 (0.779) |
| | **SPES** | −0.142 (0.146) | 0.081 (0.406) | 0.162 (0.103) | 0.217 (0.027*) |
| | | **BF** | 0.112 (0.253) | −0.038 (0.702) | −0.212 (0.032*) |
| | | | **IMF** | 0.211 (0.033*) | 0.106 (0.288) |
| | | | | **Ab (Mhp)** | 0.032 (0.752) |

GW, Goodwin lung lesion score (log-transformed); SPES, slaughterhouse pleuritis evaluation system; BF, backfat thickness; IMF, intramuscular fat; Ab, antibody; Mhp, *Mycoplasma hyopneumoniae*; App, *Actinobacillus pleuropneumoniae*.

Probability of correlation is indicated with parentheses. *** $P<0.001$; * $P<0.05$.

**Table S2.** PRR genotype–trait associations based on GLM analysis for farm A. (A) Goodwin lung lesion score; (B) SPES pleuritis lesion score; (C) backfat thickness; (D) intramuscular fat; and antibodies specific to (E) Mhp and (F) App. Sex and slaughter date were included as factors. The single *NOD1*$^{+/+}$ haplotype individual detected was excluded from the analysis. *** $P < 0.001$; ** $P < 0.01$; * $P < 0.05$.

**(A)** Goodwin lung lesion score (GW)

| Reference group | Tested group | Coefficient | SE | Confidence interval (≥95%) | | Hypothesis test | |
|---|---|---|---|---|---|---|---|
| | | | | Lower | Upper | *t* | *P* |
| Sex (Male) | Female | 0.021 | 0.102 | −0.180 | 0.222 | 0.203 | 0.839 |
| NOD1 (−/−) | +/− | −0.009 | 0.107 | −0.218 | 0.201 | −0.080 | 0.936 |
| NOD2-2197 (A/A) | A/C | −0.095 | 0.105 | −0.302 | 0.111 | −0.903 | 0.367 |
| | C/C | 0.216 | 0.349 | −0.468 | 0.901 | 0.620 | 0.536 |
| NLRP3-2906 (A/A) | A/G | −0.041 | 0.127 | −0.289 | 0.208 | −0.322 | 0.747 |
| TLR5-1205 (C/C) | C/T | −0.253 | 0.154 | −0.555 | 0.050 | −1.637 | 0.102 |
| (Date) | | 0.003 | 0.002 | −0.001 | 0.006 | 1.504 | 0.132 |
| (Intercept) | | −45.490 | 31.025 | −106.298 | 15.318 | −1.466 | 0.143 |

**(B)** Pleuritis-lesion score (SPES)

| Reference group | Tested group | Coefficient | SE | Confidence interval (≥95%) | | Hypothesis test | |
|---|---|---|---|---|---|---|---|
| | | | | Lower | Upper | *t* | *P* |
| Sex (Male) | Female | 0.013 | 0.121 | −0.225 | 0.250 | 0.104 | 0.917 |
| NOD1 (−/−) | +/− | −0.028 | 0.126 | −0.276 | 0.219 | −0.224 | 0.823 |
| NOD2-2197 (A/A) | A/C | −0.109 | 0.125 | −0.354 | 0.135 | −0.875 | 0.381 |
| | C/C | −0.313 | 0.414 | −1.124 | 0.498 | −0.757 | 0.449 |
| NLRP3-2906 (A/A) | A/G | −0.263 | 0.150 | −0.558 | 0.031 | −1.754 | 0.080 |
| TLR5-1205 (C/C) | C/T | 0.104 | 0.183 | −0.254 | 0.462 | 0.568 | 0.570 |
| (Date) | | 0.006 | 0.002 | 0.002 | 0.010 | 3.176 | 0.001** |
| (Intercept) | | −116.261 | 36.734 | −188.257 | −44.264 | −3.165 | 0.002** |

**(C)** Backfat thickness

| Reference group | Tested group | Coefficient | SE | Confidence interval (≥95%) | | Hypothesis test | |
|---|---|---|---|---|---|---|---|
| | | | | Lower | Upper | *t* | *P* |
| Sex (Male) | Female | −0.068 | 0.073 | −0.211 | 0.076 | −0.921 | 0.357 |
| NOD1 (−/−) | +/− | 0.043 | 0.076 | −0.107 | 0.193 | 0.566 | 0.572 |
| NOD2−2197 (A/A) | A/C | 0.113 | 0.075 | −0.034 | 0.261 | 1.503 | 0.133 |
| | C/C | 0.178 | 0.250 | −0.311 | 0.668 | 0.714 | 0.475 |
| NLRP3-2906 (A/A) | A/G | 0.069 | 0.091 | −0.108 | 0.247 | 0.765 | 0.444 |
| TLR5-1205 (C/C) | C/T | 0.003 | 0.110 | −0.214 | 0.219 | 0.023 | 0.981 |
| (Date) | | 0.005 | 0.001 | 0.003 | 0.007 | 4.212 | <0.001*** |
| (Intercept) | | −91.341 | 22.197 | −134.846 | −47.835 | −4.115 | <0.001*** |

**(D)** Intramuscular fat

| Reference group | Tested group | Coefficient | SE | Confidence interval (≥95%) | | Hypothesis test | |
|---|---|---|---|---|---|---|---|
| | | | | Lower | Upper | *t* | *P* |
| Sex (Male) | Female | −0.029 | 0.041 | −0.110 | 0.052 | −0.705 | 0.481 |
| NOD1 (−/−) | +/− | 0.127 | 0.043 | 0.042 | 0.211 | 2.944 | 0.003** |
| NOD2-2197 (A/A) | A/C | −0.031 | 0.042 | −0.114 | 0.052 | −0.732 | 0.464 |
| | C/C | −0.145 | 0.137 | −0.413 | 0.124 | −1.055 | 0.291 |
| NLRP3-2906 (A/A) | A/G | −0.112 | 0.051 | −0.212 | −0.013 | −2.213 | 0.027* |
| TLR5-1205 (C/C) | C/T | 0.044 | 0.062 | −0.078 | 0.165 | 0.706 | 0.480 |
| (Date) | | 0.000 | 0.001 | −0.002 | 0.001 | −0.401 | 0.689 |
| (Intercept) | | 7.322 | 12.187 | −16.565 | 31.208 | 0.601 | 0.548 |

**(E)** Mhp-specific antibodies

| Reference group | Tested group | Coefficient | SE | Confidence interval (≥95%) | | Wald′s hypothesis test | |
|---|---|---|---|---|---|---|---|
| | | | | Lower | Upper | *t* | *P* |
| Sex (Male) | Female | 0.069 | 0.031 | 0.008 | 0.131 | 2.219 | 0.026* |
| NOD1 (−/−) | +/− | 0.003 | 0.033 | −0.060 | 0.067 | 0.104 | 0.918 |
| NOD2-2197 (A/A) | A/C | −0.047 | 0.032 | −0.110 | 0.016 | −1.473 | 0.141 |
| | C/C | 0.005 | 0.107 | −0.204 | 0.214 | 0.045 | 0.964 |
| NLRP3-2906 (A/A) | A/G | −0.023 | 0.039 | −0.099 | 0.053 | −0.599 | 0.549 |
| TLR5-1205 (C/C) | C/T | −0.032 | 0.047 | −0.124 | 0.060 | −0.683 | 0.495 |
| (Date) | | 0.000 | 0.001 | −0.001 | 0.001 | 0.864 | 0.387 |
| (Intercept) | | −8.065 | 9.460 | −26.607 | 10.477 | −0.852 | 0.394 |

**(F)** App-specific antibodies

| Reference group | Tested group | Coefficient | SE | Confidence interval (≥95%) | | Hypothesis test | |
|---|---|---|---|---|---|---|---|
| | | | | Lower | Upper | *t* | *P* |
| Sex (Male) | Female | −0.876 | 2.069 | −4.931 | 3.179 | −0.424 | 0.672 |
| NOD1 (−/−) | +/− | −1.315 | 2.156 | −5.541 | 2.910 | −0.610 | 0.542 |
| NOD2-2197 (A/A) | A/C | −4.578 | 2.127 | −8.747 | −0.409 | −2.152 | 0.031* |
| | C/C | 1.812 | 7.053 | −12.011 | 15.635 | 0.257 | 0.797 |
| NLRP3-2906 (A/A) | A/G | −1.349 | 2.560 | −6.367 | 3.669 | −0.527 | 0.598 |
| TLR5-1205 (C/C) | C/T | 0.571 | 3.115 | −5.534 | 6.676 | 0.183 | 0.855 |
| (Date) | | 0.123 | 0.034 | 0.056 | 0.190 | 3.582 | <0.001*** |
| (Intercept) | | −2231.667 | 626.347 | −3459.285 | −1004.050 | −3.563 | <0.001*** |

**Table S3.** PRR genotype–trait associations based on GLM analysis for farm B. (A) Goodwin lung lesion score; (B) SPES pleuritis lesion score; (C) backfat thickness; (D) intramuscular fat; and antibodies specific to (E) Mhp and (F) App. Sex and slaughter date were included as factors. The single *NOD1*$^{+/+}$ haplotype individual detected was excluded from the analysis. *** $P < 0.001$; ** $P < 0.01$; * $P < 0.05$.

**(A)** Goodwin lung lesion score (GW)

| Reference group | Tested group | Coefficient | SE | Confidence interval (≥95%) | | Hypothesis test | |
|---|---|---|---|---|---|---|---|
| | | | | Lower | Upper | *t* | *P* |
| Sex (Male) | Female | −0.025 | 0.167 | −0.353 | 0.302 | −0.151 | 0.880 |
| NOD1 (−/−) | +/− | −0.124 | 0.211 | −0.537 | 0.289 | −0.587 | 0.557 |
| NOD2-2197 (A/A) | A/C | 0.023 | 0.192 | −0.353 | 0.399 | 0.119 | 0.905 |
| | C/C | −0.476 | 0.412 | −1.284 | 0.333 | −1.153 | 0.249 |
| NLRP3-2906 (A/A) | A/G | −0.531 | 0.234 | −0.989 | −0.072 | −2.267 | 0.023* |
| TLR5-1205 (C/C) | C/T | 0.033 | 0.264 | −0.485 | 0.550 | 0.123 | 0.902 |
| (Date) | | 0.001 | 0.003 | −0.006 | 0.008 | 0.290 | 0.772 |
| (Intercept) | | −15.760 | 61.220 | −135.749 | 104.229 | −0.257 | 0.797 |

**(B)** Pleuritis-lesion score (SPES)

| Reference group | Tested group | Coefficient | SE | Confidence interval (≥95%) | | Hypothesis test | |
|---|---|---|---|---|---|---|---|
| | | | | Lower | Upper | *t* | *P* |
| Sex (Male) | Female | 0.088 | 0.136 | −0.177 | 0.354 | 0.653 | 0.514 |
| NOD1 (−/−) | +/− | 0.112 | 0.171 | −0.223 | 0.447 | 0.654 | 0.513 |
| NOD2-2197 (A/A) | A/C | −0.325 | 0.155 | −0.630 | −0.021 | −2.092 | 0.036* |
| | C/C | −0.628 | 0.334 | −1.284 | 0.027 | −1.879 | 0.060 |
| NLRP3-2906 (A/A) | A/G | 0.059 | 0.190 | −0.313 | 0.431 | 0.313 | 0.754 |
| TLR5-1205 (C/C) | C/T | 0.652 | 0.214 | 0.233 | 1.072 | 3.049 | 0.002** |
| (Date) | | 0.008 | 0.003 | 0.003 | 0.014 | 3.079 | 0.002** |
| (Intercept) | | −152.501 | 49.635 | −249.784 | −55.218 | −3.072 | 0.002** |

**(C)** Backfat thickness

| Reference group | Tested group | Coefficient | SE | Confidence interval (≥95%) | | Hypothesis test | |
|---|---|---|---|---|---|---|---|
| | | | | Lower | Upper | *t* | *P* |
| Sex (Male) | Female | −0.143 | 0.069 | −0.278 | −0.009 | −2.086 | 0.037* |
| NOD1 (−/−) | +/− | 0.004 | 0.087 | −0.166 | 0.173 | 0.043 | 0.966 |
| NOD2-2197 (A/A) | A/C | −0.022 | 0.079 | −0.176 | 0.133 | −0.276 | 0.783 |
| | C/C | 0.011 | 0.169 | −0.321 | 0.343 | 0.065 | 0.948 |
| NLRP3-2906 (A/A) | A/G | −0.019 | 0.096 | −0.207 | 0.170 | −0.194 | 0.846 |
| TLR5-1205 (C/C) | C/T | −0.139 | 0.108 | −0.351 | 0.074 | −1.278 | 0.201 |
| (Date) | | −0.005 | 0.001 | −0.008 | −0.003 | −3.864 | <0.001*** |
| (Intercept) | | 99.278 | 25.136 | 50.012 | 148.544 | 3.950 | <0.001*** |

**(D)** Intramuscular fat

| Reference group | Tested group | Coefficient | SE | Confidence interval (≥95%) | | Hypothesis test | |
|---|---|---|---|---|---|---|---|
| | | | | Lower | Upper | *t* | *P* |
| Sex (Male) | Female | 0.019 | 0.041 | −0.061 | 0.099 | 0.464 | 0.643 |
| NOD1 (−/−) | +/− | 0.071 | 0.051 | −0.029 | 0.172 | 1.392 | 0.164 |
| NOD2-2197 (A/A) | A/C | −0.018 | 0.047 | −0.109 | 0.074 | −0.381 | 0.703 |
| | C/C | 0.046 | 0.100 | −0.150 | 0.243 | 0.463 | 0.643 |
| NLRP3-2906 (A/A) | A/G | 0.038 | 0.057 | −0.074 | 0.149 | 0.661 | 0.509 |
| TLR5-1205 (C/C) | C/T | 0.138 | 0.064 | 0.012 | 0.264 | 2.151 | 0.031* |
| (Date) | | 0.000 | 0.001 | −0.002 | 0.001 | −0.572 | 0.567 |
| (Intercept) | | 11.036 | 14.890 | −18.148 | 40.221 | 0.741 | 0.459 |

**(E)** Mhp-specific antibodies

| Reference group | Tested group | Coefficient | SE | Confidence interval (≥95%) | | Hypothesis test | |
|---|---|---|---|---|---|---|---|
| | | | | Lower | Upper | *t* | *P* |
| Sex (Male) | Female | 0.193 | 0.082 | 0.032 | 0.354 | 2.351 | 0.019* |
| NOD1 (−/−) | +/− | −0.027 | 0.103 | −0.228 | 0.175 | −0.259 | 0.796 |
| NOD2-2197 (A/A) | A/C | −0.040 | 0.095 | −0.225 | 0.146 | −0.420 | 0.675 |
| | C/C | −0.271 | 0.200 | −0.662 | 0.121 | −1.355 | 0.175 |
| NLRP3-2906 (A/A) | A/G | −0.201 | 0.118 | −0.432 | 0.030 | −1.702 | 0.089 |
| TLR5-1205 (C/C) | C/T | 0.208 | 0.128 | −0.042 | 0.458 | 1.629 | 0.103 |
| (Date) | | −0.004 | 0.002 | −0.007 | −0.001 | −2.330 | 0.020* |
| (Intercept) | | 71.811 | 30.382 | 12.264 | 131.358 | 2.364 | 0.018* |

**(F)** App-specific antibodies

| Reference group | Tested group | Coefficient | SE | Confidence interval (≥95%) | | Hypothesis test | |
|---|---|---|---|---|---|---|---|
| | | | | Lower | Upper | *t* | *P* |
| Sex (Male) | Female | −6.779 | 4.806 | −16.198 | 2.640 | −1.411 | 0.158 |
| NOD1 (−/−) | +/− | 0.099 | 6.022 | −11.704 | 11.902 | 0.016 | 0.987 |
| NOD2-2197 (A/A) | A/C | 3.545 | 5.546 | −7.324 | 14.414 | 0.639 | 0.523 |
| | C/C | −5.266 | 11.701 | −28.200 | 17.668 | −0.450 | 0.653 |
| NLRP3-2906 (A/A) | A/G | −14.263 | 6.917 | −27.819 | −0.707 | −2.062 | 0.039* |
| TLR5-1205 (C/C) | C/T | 5.329 | 7.468 | −9.308 | 19.965 | 0.714 | 0.475 |
| (Date) | | 0.103 | 0.098 | −0.090 | 0.295 | 1.048 | 0.295 |
| (Intercept) | | −1851.829 | 1779.107 | −5338.814 | 1635.156 | −1.041 | 0.298 |
